# Undergraduate medical research in the West Bank, Palestine: a cross-sectional analysis of student knowledge, attitudes, barriers, and engagement across five universities

**DOI:** 10.3389/fmed.2026.1803680

**Published:** 2026-05-01

**Authors:** Azzam Zrineh, Rami Akwan, Roaa Darawsheh, Hebatallah Qawasmeh, Marwa Zahdeh, Yaman Abu Sarrees, Ali Shakhshir, Bader Awesat, Wisam Mustafa, Islam Ishnawer, Hamza Karmi, Abdallah Alwawi

**Affiliations:** 1 Faculty of Medicine, Al-Quds University, Jerusalem, Palestine; 2 Faculty of Medicine, Syrian Private University, Damascus, Syria; 3 Faculty of Medicine and Allied Medical Sciences, An-Najah National University, Nablus, Palestine; 4 Faculty of Medicine, Palestine Polytechnic University, Hebron, Palestine; 5 Faculty of Medicine, Arab American University, Jenin, Palestine; 6 Faculty of Medicine, Hebron University, Hebron, Palestine; 7 Faculty of Health Professions, Al-Quds University, Jerusalem, Palestine

**Keywords:** medical research, medical students, Palestine, research attitudes, research barriers, research knowledge, research participation, undergraduate medical education

## Abstract

**Background:**

Medical research integration within undergraduate education is critical for developing scientifically literate physicians capable of evidence-based practice. However, profound disparities persist globally, particularly in low- and middle-income countries where students encounter substantial barriers including inadequate infrastructure, limited funding, and insufficient mentorship. Palestinian medical students navigate additional unique challenges, including political instability, movement restrictions, and frequent academic disruptions. This study provides a comprehensive multi-institutional assessment of research knowledge, attitudes, barriers, and engagement among Palestinian medical students in the West Bank.

**Methods:**

A cross-sectional study was conducted among 625 undergraduate medical students (second to sixth year) from five Palestinian universities. Data were collected using a structured questionnaire adapted from previously validated instruments in the literature, comprising sections assessing demographics, research knowledge, attitudes, perceived barriers, and research practices. All data were analyzed using the IBM SPSS statistical package (version 25).

**Results:**

Nearly half of students (49.1%) demonstrated poor research knowledge, 33% fair knowledge, and only 17.9% satisfactory knowledge. Despite these deficits, students showed remarkably positive attitudes toward research (median: 70/93) and recognized its professional value (median: 25/27). Research participation was moderate (45.9%), but scholarly output remained limited, with 74.9% never publishing any work. Students reported high barrier burden (median: 75/96), with primary obstacles including lack of funding (64%), insufficient time (62.1%), limited statistical knowledge (60.5%), and universities prioritizing education over research (60.5%). Research methodology training, parental education, university affiliation, and academic year significantly influenced knowledge scores and participation rates.

**Conclusion:**

Palestinian medical students recognize research value but face substantial knowledge gaps and systemic barriers that limit engagement and scholarly dissemination. Addressing these challenges requires longitudinal curriculum integration, structured mentorship programs, dedicated research funding, and institutional support to develop a research-literate healthcare workforce capable of addressing local and regional health challenges.

## 1 Introduction

Medical research serves as the cornerstone of evidence-based medicine and healthcare innovation, driving improvements in disease prevention, diagnosis, and treatment (1). Beyond its direct contribution to clinical practice, research integration within medical education has emerged as a critical component in developing competent, scientifically literate physicians capable of critical appraisal, methodological rigor, and lifelong learning (2). Early exposure to research during undergraduate medical training cultivates essential competencies, including analytical thinking, scientific reasoning, and evidence synthesis skills that extend beyond academic benefits to enhance clinical decision-making throughout professional careers (3–5). Despite widespread recognition of research’s pivotal role in medical education and practice, profound disparities persist globally in research engagement, infrastructure, and productivity, particularly between high-income countries and low- and middle-income countries (LMICs) (6–9). Students in resource-limited settings frequently encounter substantial barriers, including inadequate infrastructure, limited funding, and insufficient mentorship, which are further intensified by demanding academic workloads, time constraints, and restricted access to research databases (10–14). Consequently, medical students and graduates from such settings may lack essential research competencies, perpetuating a cycle of limited research productivity and reduced contribution to the global scientific knowledge base.

Within the Arab world, many studies have investigated the research landscape among medical students. A landmark multi-country investigation provided important evidence in this regard (15). The study involving 2,989 medical students across six Arab countries revealed that 91.6% exhibited poor research knowledge despite maintaining positive attitudes toward research, with principal barriers including lack of access to laboratory equipment (68.1%), prioritization of education over research (66.8%), and time constraints due to educational demands (66.1%). Additionally, only one-third of students reported participation in research projects, primarily cross-sectional studies and case reports, with a low average of publications per participating student, highlighting the substantial gap between participation and successful research dissemination (15). Similar findings were reported among Sudanese and Moroccan medical students, with comparable low knowledge scales, a positive stance toward research, and predominant barriers including insufficient funding, limited time, and inadequate laboratory facilities (16, 17). Collectively, these studies from across the Arabic region demonstrate a consistent pattern: medical students recognize the value of research, yet face multiple systemic barriers that prevent the translation of this positive attitude into meaningful research engagement.

Within a challenging regional context, Palestine presents a uniquely complex situation characterized by compounding adversities that profoundly impact medical education and research involvement. Palestinian medical students navigate an educational environment marked by political instability, severe movement restrictions affecting access to clinical training sites and academic facilities, substantial economic constraints, and frequent disruptions to academic continuity. This constellation of obstacles establishes an exceptionally challenging environment for cultivating research engagement. However, it also underscores the importance of supporting the development of local research to address Palestine’s specific health needs and contribute meaningfully to regional and global health knowledge.

Therefore, understanding the current state of research among Palestinian medical students is crucial for developing targeted interventions to strengthen research culture and capacity. Our study adds to the limited body of literature on medical research education in conflict-affected and resource-constrained settings. It provides a comprehensive multi-institutional assessment evaluating students’ research knowledge, attitudes, barriers, and engagement across major Palestinian universities in the West Bank.

## 2 Materials and methods

### 2.1 Study design and setting

This cross-sectional study was conducted among undergraduate medical students enrolled in five Palestinian universities in the West Bank: Al-Quds University (Jerusalem), An-Najah National University (Nablus), Palestine Polytechnic University (Hebron), Hebron University (Hebron), and Arab American University (Jenin). These institutions represent major medical education providers in Palestine. The study was conducted from September 17 to November 1, 2025. Reporting for this study was conducted in line with the Strengthening the Reporting of Observational Studies in Epidemiology (STROBE) guidelines.

### 2.2 Study population and sampling

The target population consisted of undergraduate medical students aged 18 + (second to sixth year) from the five participating universities. First-year students were excluded due to limited research exposure. Postgraduate students, internship doctors, and residents were also excluded.

Convenience sampling through social media platforms was employed. Sample size calculation was performed using OpenEpi (18) with 50% expected frequency, 95% confidence level, and 5% margin of error, yielding a minimum required sample of 362 participants. However, to enhance statistical power and secure adequate representation across universities and academic levels, we aimed to increase the sample size to a minimum of 500 participants.

### 2.3 Data collection instrument

The questionnaire was adapted with permission from validated instruments used in previous research, specifically from the study by Mohamedzain et al. (16) conducted in Sudan. Key adaptations included contextualizing demographic items to the Palestinian educational and socioeconomic context. No substantive modifications were made to the core knowledge, attitude, or barrier scales, thereby preserving the validated psychometric properties of the original instruments. The adapted questionnaire was reviewed by two medical education experts and pilot-tested among 30 students to ensure clarity, relevance, and cultural appropriateness. The questionnaire was administered in English, as it is the language of instruction in Palestinian medical schools. Our analyses indicated adequate internal consistency with Cronbach’s *α* > 0.7 in respective sections.

The questionnaire comprised five sections. The first section collected demographic information, such as age, gender, university, and academic year, among others. The second section assessed research knowledge through eight multiple-choice questions evaluating basic research concepts (Cronbach’s *α* = 0.813), with correct responses scored as one point and incorrect or “I don’t know” responses scored as zero. Knowledge levels were categorized as poor (less than 50%), fair (50 to 75%), and satisfactory (more than 75%). The third section was based on the Attitude Toward Research (ATR) Scale (19) using 31 items on a 3-point Likert scale. For positively worded statements, scoring was (agree = 3, uncertain/I do not know = 2, disagree = 1), while for negative statements, scoring was reversed. This section included the following subscales: research usefulness in professional life (maximum score: 27), research anxiety, which captures the negative feelings of stress and worry that arise in relation to engaging in research activities and participating in courses related to research (maximum score: 24), positive attitudes (maximum score: 21), research relevance to life (maximum score: 12), and perceived difficulty in research (maximum score: 9) (Overall Cronbach’s *α* = 0.860).

The fourth section addressed perceived barriers to research through 32 items on a 3-point Likert scale (agree = 3, uncertain, I do not know = 2, disagree = 1) (Cronbach’s α = 0.937). The total burden score was categorized into low (< 50%), moderate (50–75%), and high (>75%). The final section examined research practices using eight questions that assessed prior participation in research activities, number of publications, and presentations. Research output was categorized into three groups: None, 1–2, and ≥3 activities. These cutoffs were selected to reflect meaningful distinctions in research engagement within a resource-constrained setting.

### 2.4 Data collection procedure

Data were collected using an anonymous, self-administered online questionnaire via Google Forms. The survey was distributed through multiple platforms, including university Facebook groups, WhatsApp groups, and Telegram channels. To minimize the risk of duplicate responses, the Google Forms survey was configured to limit submissions to one response per Google account. Additionally, response patterns and timestamps were examined during data cleaning to detect potential duplicate entries.

### 2.5 Statistical analysis

All data were analyzed using the IBM SPSS statistical package (version 25), with a two-sided *p* value < 0.05 being considered statistically significant. Normality of continuous data was assessed using the Kolmogorov–Smirnov test and plots visualization, which indicated non-normal distribution. Continuous variables were reported using median and interquartile range (IQR; Q1–Q3), while categorical variables were reported using frequencies and percentages.

Comparisons of scores across demographic variables were conducted using the Mann–Whitney U test for two-group comparisons, and the Kruskal-Wallis test for more than two groups. For each group comparison, median and IQR (Q1–Q3), mean ranks, and *p*-values were reported. Whenever the Kruskal-Wallis test was statistically significant, *post hoc* pairwise comparisons were performed using the Dunn test, and Bonferroni-adjusted *p* values were reported.

Associations between categorical variables were examined using the Chi-square test. For significant multi-group comparisons, post hoc analysis was conducted by examining adjusted residuals (z-scores) to identify cells contributing to the association. Residuals exceeding the absolute z-score corresponding to the Bonferroni-adjusted alpha were considered significant, and a *p*-value for each significant cell was calculated from the squared residuals using SPSS. This was based on methodological recommendations by García-pérez et al. (20). To identify predictors of previous research participation, multivariable logistic regression was performed. The dependent variable was previous participation in a research project (no/yes). Independent variables included selected demographic factors, as well as knowledge, total attitudes, and barrier burden scores, chosen based on theoretical relevance to research engagement. Results are reported as adjusted odds ratios (aOR) with 95% confidence intervals (CI).

### 2.6 Ethical considerations

Ethical approval was obtained from Al-Quds University Research Ethics Committee (Ref No: 587/REC/2025). Before accessing the questionnaire, participants were presented with a section that provided clear and comprehensive information regarding the study’s aims, their voluntary participation, and data confidentiality, with electronic informed consent obtained before proceeding to the questionnaire. All collected data were anonymized and stored securely, with access restricted exclusively to the research team.

## 3 Results

A total of 625 undergraduate medical students from five Palestinian universities participated in the study. The median age was 21 years, with males representing 52.6% of the sample. The largest group was from An-Najah National University, followed closely by Palestine Polytechnic University and Al-Quds University. Most were in their third (26.1%) and fifth (22.9%) years of study. Regarding parents’ education, the most common level was a bachelor’s degree for both fathers (44.2%) and mothers (54%). Most respondents (81%) owned a personal computer or laptop, while 14.4% shared one with family, 2.9% relied on public facilities, and 1.7% had no computer access. Additionally, 78.2% had completed a research methodology course at university. Further details are available in Table 1.

**Table 1:** Demographic and educational characteristics of participating undergraduate medical students (*N* = 625).

Baseline variable		*N* (%)
Age: Median (Q1 – Q3)	21 [20–22]	
Gender	Female	296 (47.4)
	Male	329 (52.6)
Residence	Rural	259 (41.5)
	Urban	332 (53.1)
	Refugee camp	34 (5.4)
University	Al-Quds University	143 (22.8)
	An-Najah National University	146 (23.4)
	Hebron University	85 (13.6)
	Polytechnic University	145 (23.2)
	Arab American University	106 (17)
Academic year	Second	98 (15.7)
	Third	163 (26.1)
	Fourth	105 (16.8)
	Fifth	143 (22.9)
	Sixth	116 (18.5)
Father’s educational status	No formal education/Can read and write only	20 (3.2)
	Primary or/and Preparatory education	44 (7)
	Secondary education/Tawjihi	138 (22.1)
	Bachelor’s degree	276 (44.2)
	Postgraduate degrees (Master’s/PhD)	147 (23.5)
Mother’s educational status	No formal education/Can read and write only	19 (3)
	Primary or/and Preparatory education	34 (5.4)
	Secondary education/Tawjihi	152 (24.3)
	Bachelor’s degree	337 (54)
	Postgraduate degrees (Master’s/PhD)	83 (13.3)
Monthly income	Less than 4,000 NIS	0
	4,000–6,000 NIS	257 (41)
	6,001–8,000 NIS	94 (15)
	More than 8,000 NIS	137 (22)
	Prefer not to answer	137 (22)
Access to a computer or a laptop	Personal device	506 (81)
	Shared family device	90 (14.4)
	Usage of the university/public facilities’ computer	18 (2.9)
	No access to a computer or a laptop	11 (1.7)
Studied research methodology at the university	Yes	489 (78.2)
	No	136 (21.8)

### 3.1 Undergraduates’ knowledge of research

The median total knowledge score was 4 out of 8 (IQR: 2–6). When categorized, 49.1% of students had poor knowledge, 33% had fair knowledge, and 17.9% had satisfactory knowledge about research.

### 3.2 Undergraduates’ attitudes towards research

Participants generally viewed research positively, reflected in a high overall median score of 70 out of 93 (IQR: 63–77). They demonstrated particularly high scores in domains of research usefulness in professional career with a median score of 25 (IQR: 22–26) out of 27, and in positive attitudes towards research with a median score of 18 (IQR: 14–21) out of 21. Conversely, they viewed research to be moderately anxiety-inducing and somewhat difficult, with median scores of 15 (IQR: 12–18) out of 24 and 6 (IQR: 5–7) out of 9, respectively, but still recognized its relevance to daily life, scoring a median of 9 (IQR: 8–10) out of 12.

### 3.3 Barriers faced by medical undergraduates in research

The most commonly reported research barriers among Palestinian medical students were insufficient funding (64%), the lack of time to conduct research due to heavy academic responsibilities (62.1%), and the perception that their universities prioritize education over research (60.5%), with an equal proportion (60.5%) reporting limited familiarity with statistical analysis. Additionally, over half of the participants (55.5%) felt their research skills were inadequate, and 54.7% lacked the skills needed to submit their articles to academic journals. Nonetheless, only 35.8% expressed disinterest in conducting research. The frequencies of all barriers are detailed in Supplementary material 1.

Overall, the barrier burden score had a median of 75 (IQR: 65–85) out of 96. Nearly half of the participants (49.8%) experienced a high burden (greater than 75%), while 45.6% experienced a moderate burden (between 50 and 75%), and only 4.6% had a low burden score (<50%).

### 3.4 Undergraduates’ practices towards research

Among all participants, 44% had attended a research methodology workshop, while 45.9% reported participating in previous research projects. Approximately two-thirds of these students (67.6%) had been involved in one to two projects, whereas 32.4% had participated in three or more. In terms of publication experience, 74.9% had not published any work, 18.8% had published one to two articles, and only 6.3% had three or more publications. Additionally, 80.2% of participants had no prior experience with poster presentation, 14.7% had presented one to two posters, and 5.1% had presented three or more. Oral presentation followed the same pattern, with 73.3% of students having no previous experience, 19% presenting one to two times, and 7.7% delivering three or more presentations.

Among participants who had previously conducted research (*n* = 287), the most commonly reported tasks were writing proposals and reviewing literature (62.4% each). This was followed by contributing to the research concept (47%), performing data entry (43.6%), and manuscript writing (37.6%). Fewer participants reported performing statistical analyses (26.5%) or contributing to the execution of research (22%). Additionally, most participants were involved in primary observational studies, particularly cross-sectional studies (42.2%) and case reports (35.5%), followed by retrospective clinical studies (28.6%). Secondary research was less common, with 22.6% reporting involvement in review articles and meta-analyses. Notably, prospective clinical studies and clinical trials had the lowest participation rates at 8.7 and 7%, respectively, whereas 15.3% of students participated in basic study designs.

### 3.5 Group comparison of knowledge score, attitudes` subscales, and barriers burden score based on demographics

**Figure 1:**
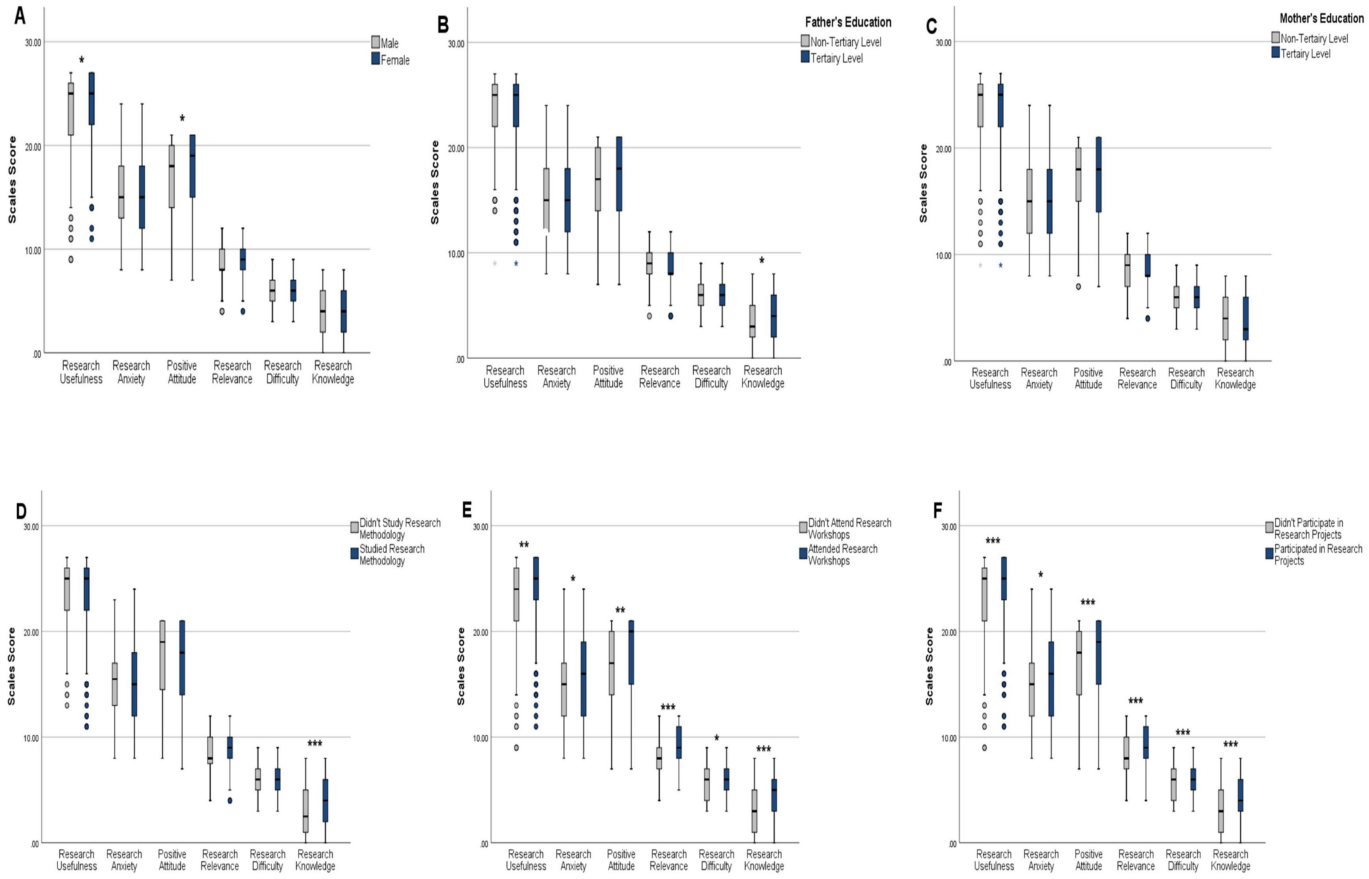
Comparison of knowledge score and attitude subscales scores across sociodemographic and research activities (*N* = 625). **(A)** Gender, **(B)** father’s education, **(C)** mother’s education, **(D)** studying research methodology at the university, **(E)** attending a research workshop, **(F)** participating in research projects. Scores were compared between groups using the Mann–Whitney U test. Boxes represent the interquartile range (Q1–Q3), horizontal lines indicate the median, and whiskers extend to 1.5 times the interquartile range. Significance levels: *** *p* < 0.001, ** *p* < 0.01, * *p* < 0.05.

Knowledge scores were significantly higher among students who attended courses in research methodology at the university, enrolled in a research workshop, or participated in a research project (*p* value < 0.001 for each). Higher knowledge scores were also observed among students whose fathers held a bachelor’s degree or higher (*p* value = 0.034). Score comparisons across sociodemographic variables and research activities are presented in Figure 1. Additionally, knowledge scores varied across universities and academic years, with *post hoc* analysis revealing a notable difference between An-Najah University and Arab American University (median: 4 vs. 3, adjusted *p* value = 0.024) and between fourth-year and second-year students (median: 5 vs. 3, adjusted *p* value = 0.013).

The positive attitudes score also differed across universities and academic years, with students from Arab American University scoring higher than those from Al Quds University (adjusted *p* value < 0.001), Polytechnic University (adjusted *p* value = 0.003), and Hebron University (adjusted *p* value = 0.02). Moreover, a significant association was noted between fourth-year and fifth-year students, with the latter having lower median scores (adjusted *p* value = 0.013).

Regarding the anxiety score, students from Arab American University expressed higher anxiety related to conducting research compared to students from Al-Quds and Polytechnic University (adjusted *p* value <0.05 for each pairwise comparison). Fourth-year students also exhibited higher anxiety scores than sixth-year students (adjusted *p* value = 0.035). Students from Al-Najah University perceived research as more difficult to conduct compared to Al-Quds University students (adjusted *p* value = 0.006).

Barrier burden scores varied across academic years, with fourth-year students reporting a lower median than third-year and sixth-year students (medians: 71 vs. 78 vs. 78, adjusted *p* value = 0.03 and 0.012, respectively).

Finally, all attitude subscales scores were significantly higher for participants who had previously enrolled in a research workshop or conducted a research project (Figure 1). Detailed comparisons of scores based on university affiliation and academic year are presented in Tables 2, 3. Additional analyses of score differences across other demographic variables are provided in Supplementary material 1.

**Table 2:** Comparison of knowledge, attitude subscale, and barrier burden scores based on university affiliation (*N* = 625).

Score	Al-Quds University	An-Najah University	Hebron University	Polytechnic University	Arab American University	*P*-value
Knowledge score	3 [2–5]	4 [2–6]	4 [2–7]	3 [2–6]	3 [1–6]	0.007*
	*293.33*	*345.54*	*346.21*	*307.20*	*276.03*	
Research usefulness	25 [21–26]	25 [22–26.25]	24 [21.50–26]	25 [21.50–26]	26 [23–27]	0.056
	*300.45*	*325.17*	*285.01*	*300.98*	*352.05*	
Anxiety score	15 [11–18]	15 [13–18]	15 [11–18]	15 [12–17]	16 [13.75–19]	0.012*
	*295.56*	*327.05*	*298.63*	*289.19*	*361.28*	
Positive attitude	17 [14–20]	19 [14–21]	17 [14–20]	18 [14–20]	20 [15.75–21]	<0.001*
	*280.54*	*333.30*	*293.25*	*291.88*	*373.56*	
Research relevance	8 [7–10]	9 [8–10]	9 [8–10]	8 [7–10]	9 [8–11]	0.165
	*301.42*	*325.95*	*302.72*	*294.58*	*344.23*	
Difficulty score	6 [4–7]	6 [5–7]	6 [4–7]	6 [5–7]	6 [5–7]	0.006*
	*269.63*	*341.40*	*299.46*	*320.82*	*332.56*	
Barriers burden score	77 [68–85]	76 [65–85]	77 [64–87]	76 [65–85]	73 [64–81.25]	0.329
	*330.94*	*310.05*	*317.09*	*318.10*	*282.60*	

For each score, the first row presents Median [Q1 – Q3] and the second row (in italics) presents Mean Rank. *P*-values derived from the Kruskal-Wallis test. Bold values indicate statistical significance (* *p* < 0.05).

**Table 3:** Comparison of knowledge, attitude subscale, and barrier burden scores based on academic year (*N* = 625).

Score	Second year	Third year	Fourth year	Fifth year	Sixth year	*P*-value
Knowledge score	3 [1.75–5]	3 [2–6]	5 [2–7]	3 [2–6]	4 [2–6]	0.019*
	*270.12*	*306.65*	*351.24*	*307.80*	*329.94*	
Research usefulness	24 [20–26]	25 [23–26]	25 [22–27]	25 [22–26]	25 [22–26]	0.194
	*281.79*	*331.57*	*329.78*	*305.00*	*307.95*	
Anxiety score	15 [12–17]	15 [13–18]	16 [14–19]	15 [11–18]	15 [11–18]	0.022*
	*293.19*	*319.19*	*361.78*	*301.64*	*290.89*	
Positive attitude	17 [14–20]	19 [15–21]	20 [15–21]	17 [14–20]	17.50 [14–20]	0.004*
	*290.75*	*336.85*	*354.69*	*280.73*	*300.33*	
Research relevance	8 [8–10]	9 [7–10]	9 [8–10]	8 [8–10]	9 [8–10]	0.058
	*295.58*	*305.75*	*355.98*	*294.67*	*321.60*	
Difficulty score	6 [4–7]	6 [5–7]	6 [5–7]	6 [5–7]	6 [4–7]	0.099
	*295.83*	*311.60*	*354.66*	*309.65*	*295.89*	
Barriers burden score	74.50 [65–82]	78 [66–88]	71 [63–82]	73 [64–85]	78 [69–86]	0.006*
	*303.91*	*335.95*	*268.93*	*297.31*	*347.66*	

For each score, the first row presents Median [Q1 – Q3] and the second row (in italics) presents Mean Rank. *P*-values derived from the Kruskal-Wallis test. Bold values indicate statistical significance (*p < 0.05).

### 3.6 Group comparison of practice patterns based on demographics

Participation in research studies was significantly associated with male gender, higher parental education (tertiary level for both parents), and completion of a research methodology course. Enrollment in research workshops was significantly associated with completion of a methodology course at the university.

University affiliation significantly influenced research involvement and workshop attendance. *Post hoc* analysis revealed that students from An-Najah University and Polytechnic University contributed significantly to research participation, while students from the Arab American University contributed significantly to workshop training (all *p* values < 0.001).

Regarding academic years, there was also a significant association with research participation and enrollment in training (all *p* values < 0.001). Post hoc analysis found that third and sixth-year students contributed to these associations, with sixth-year students showing the highest involvement (83.6%) and training enrollment (60.3%), while third-year students had lower-than-expected participation (28.8%) and training enrollment (33.7%). Second-year students exhibited a low research participation rate relative to typical levels (23.5%). Full details are found in Tables 4, 5.

**Table 4:** Comparison of prior research participation (yes/no) across demographic and educational characteristics (*N* = 625).

Variable		Yes (*n* = 287)	No (*n* = 338)	*P*-value
Gender	Female	115 (38.9%)	181 (61.1%)	0.001*
	Male	172 (52.3%)	157 (47.7%)	
Residence	Urban	154 (46.4%)	178 (53.6%)	0.260
	Rural	122 (47.1%)	137 (52.9%)	
	Refugee camp	11 (32.4%)	23 (67.6%)	
Father’s educational status	Tertiary level education	212 (50.1%)	211 (49.9%)	0.002*
	Non-tertiary level education	75 (37.1%)	127 (62.9%)	
Mother’s educational status	Tertiary level education	205 (48.8%)	215 (51.2%)	0.038*
	Non-tertiary level education	82 (40%)	123 (60%)	
Studied research methodology at the university	Yes	254 (51.9%)	235 (48.1%)	<0.001*
	No	33 (24.3%)	103 (75.7%)	
University	Al-Quds University	79 (55.2%)	64 (44.8%)	<0.001*
	An-Najah University	85 (58.2%)	61 (41.8%)	
	Hebron University	30 (35.3%)	55 (64.7%)	
	Polytechnic University	48 (33.1%)	97 (66.9%)	
	Arab American University	45 (42.5%)	61 (57.5%)	
Academic year	Second	23 (23.5%)	75 (76.5%)	<0.001*
	Third	47 (28.8%)	116 (71.2%)	
	Fourth	43 (41%)	62 (59%)	
	Fifth	77 (53.8%)	66 (46.2%)	
	Sixth	97 (83.6%)	19 (16.4%)	

*P*-values calculated using the Chi-square test. Bold values indicate statistical significance (*p < 0.05).

**Table 5:** Comparison of prior workshops enrollment (yes/no) across demographic and educational characteristics (*N* = 625).

Variable		Yes (*n* = 275)	No (*n* = 350)	*p*-value
Gender	Female	139 (47%)	157 (53%)	0.157
	Male	136 (41.3%)	193 (58.7%)	
Residence	Urban	147 (44.3%)	185 (55.7%)	0.202
	Rural	118 (45.6%)	141 (54.4%)	
	Refugee camp	10 (29.4%)	24 (70.6%)	
Father’s educational status	Tertiary level education	191 (45.2%)	232 (54.8%)	0.400
	Non-Tertiary level education	84 (41.6%)	118 (58.4%)	
Mother’s educational status	Tertiary level education	190 (45.2%)	230 (54.8%)	0.372
	Non-Tertiary level education	85 (41.5%)	120 (58.5%)	
Studied research methodology at the university	Yes	238 (48.7%)	251 (51.3%)	<0.001*
	No	37 (27.2%)	99 (72.8%)	
University	Al-Quds University	54 (37.8%)	89 (62.2%)	0.002*
	An-Najah University	70 (47.9%)	76 (52.1%)	
	Hebron University	34 (40%)	51 (60%)	
	Polytechnic University	54 (37.2%)	91 (62.8%)	
	Arab American University	63 (59.4%)	43 (40.6%)	
Academic year	Second	33 (33.7%)	65 (66.3%)	<0.001*
	Third	55 (33.7%)	108 (66.3%)	
	Fourth	49 (46.7%)	56 (53.3%)	
	Fifth	68 (47.6%)	75 (52.4%)	
	Sixth	70 (60.3%)	46 (39.7%)	

*P*-values calculated using the Chi-square test. Bold values indicate statistical significance (* *p* < 0.05).

### 3.7 Factors associated with research projects participation: multivariable analysis

Multivariable logistic regression was performed to identify independent predictors of research participation (Table 6). The overall model was statistically significant (*p* < 0.001), explaining 37.6% of the variance in participation.

**Table 6:** Multivariable logistic regression analysis of factors associated with previous participation in research projects.

Variable	Adjusted OR (95% CI)	*P*-value
Gender (female)	0.809 (0.545–1.201)	0.293
Father’s education (tertiary level)	1.294 (0.830–2.020)	0.256
Mother’s education (tertiary level)	1.133 (0.736–1.743)	0.571
University (overall)	–	<0.001*
An-Najah University	0.511 (0.265–0.986)	0.045*
Hebron University	0.260 (0.131–0.518)	<0.001*
Polytechnic University	0.221 (0.118–0.414)	<0.001*
Arab American University	0.303 (0.156–0.588)	<0.001*
Academic year (overall)	–	<0.001*
Third year	1.139 (0.598–2.167)	0.693
Fourth year	2.222 (1.058–4.667)	0.035*
Fifth year	4.835 (2.416–9.678)	<0.001*
Sixth year	21.766 (9.496–49.890)	<0.001*
Studied research methodology at the university (yes)	1.283 (0.744–2.215)	0.370
Attitudes total score	1.043 (1.021–1.065)	<0.001*
Barrier burden	0.992 (0.978–1.006)	0.281
Knowledge total score	1.166 (1.072–1.269)	<0.001*

The dependent variable is previous participation in research projects (No/Yes). Reference categories for categorical variables are: (Gender: Male, university: Al-Quds University, Academic Year: Second year, Father’s/Mother’s education: Non-tertiary level education, Studied research methodology: No). Nagelkerke *R*^2^ = 0.376, χ^2^ (15) = 206.218. Bold values indicate statistical significance (* *p* < 0.05).

Academic year was the most prominent predictor. Compared to second-year students, the odds of participating in research projects increased progressively in the later years of study, peaking in the sixth year (aOR = 21.77, 95% CI: 9.50–49.89, *p* < 0.001). University affiliation was also a significant predictor overall (*p* < 0.001). Compared to Al-Quds University, students from all other universities had significantly lower odds of participation, with those at Polytechnic University showing the lowest odds (aOR = 0.22, 95% CI: 0.12–0.41, *p* < 0.001). Additionally, higher knowledge and attitude scores were independent predictors; with every one-unit increase in these scores, the odds of participation rose by 17 and 4%, respectively. In contrast, gender, parental education, prior completion of a research methodology course, and perceived barriers did not significantly influence participation after adjusting for other variables.

## 4 Discussion

This study presents a multi-institutional assessment of the research landscape among medical students in the West Bank, Palestine, building on and extending regional evidence. While the overall pattern of poor knowledge and positive attitudes is consistent with regional findings, the Palestinian context introduces uniquely compounding factors: chronic military occupation imposes severe movement restrictions affecting access to academic facilities and clinical training sites, recurrent armed conflict causes prolonged academic disruptions, and inter-institutional collaboration is constrained by geographic fragmentation of the West Bank. These factors create a distinct layer of barriers not captured in studies from politically stable LMICs.

Our observations closely parallel findings from the broader Arab region, where Assar et al. (15) documented that 91.6% of medical students across six Arab countries (Egypt, Algeria, Sudan, Jordan, Syria, and Palestine) exhibited poor research knowledge. Additionally, in Morocco, Chenfouh et al. (17) found a relatively low knowledge base among medical students. Similarly, the Sudanese study by Mohamedzain et al. (16) reported that 92% of medical students demonstrated poor research knowledge despite holding positive attitudes. In Egypt, Orebi et al. (12) similarly found that only nearly half of medical students had acceptable research knowledge. This consistency across studies from multiple Arab countries conveys the presence of systemic challenges in research education at a regional level rather than deficiencies attributable solely to individual institutions.

The observed knowledge deficits were evident in students’ limited competencies in fundamental research skills. Over half of the students (56%) were unfamiliar with different research study designs, and 60.5% reported limited understanding of statistical analysis. This highlights gaps in students’ methodological understanding, which may account for the moderate research participation and low publication rates observed. Notably, our study identified significant associations between research knowledge and several demographic factors. Students from An-Najah and Hebron universities achieved higher scores than those from other institutions. Overall, knowledge was generally higher among clinical years students compared to preclinical years, with a significant difference between second-year and fourth-year students. This suggests that progression into higher academic years and increased clinical exposure contribute to research literacy development, a trend consistent with a recent Jordanian study by Abusamak et al. (13), who found that students’ research knowledge increases significantly as they move into higher clinical years (*p* < 0.001). Moreover, we found that students who had attended research methodology workshops or participated in previous research projects exhibited markedly higher knowledge scores (*p* < 0.001). Taken together, these findings underscore that passive curricular exposure alone may be insufficient, and that structured, hands-on research experiences are critical for bridging knowledge gaps.

On the other hand, despite substantial knowledge deficits, students exhibited remarkably positive attitudes toward research. They highly recognized the value of research for their professional careers (median: 25 out of 27) and daily lives (median: 9 out of 12). This mirrors findings documented in recent regional studies, including studies among medical students in Sudan, Morocco, and Yemen (16, 17, 21). Notably, Turk et al. (22) showed among Syrian medical students that positive attitudes were present even among those who had never participated in research projects, suggesting that favorable attitudes alone are insufficient to drive research engagement without adequate knowledge and skills. This paradox has significant implications, as efforts that focus solely on motivating students to engage in research are unlikely to be effective unless they are supported by structured skill development that equips students with the methodological abilities needed to transform positive attitudes into active research involvement.

Students reported moderate levels of research-related anxiety (median: 15/24), reflecting apprehension toward the research process, including fear of making errors and feeling overwhelmed, rather than indicating a clinically significant anxiety disorder. This finding aligns with the broader concept of research anxiety documented in educational literature, where students experience a sense of worry or apprehension associated with conducting research tasks (23).

Participation in research projects was moderate (45.9%), with approximately two-thirds of students (67.6%) participating in one to two projects. However, scholarly output remained limited. Three-quarters of students (74.9%) never published any work, while 80.2% reported no poster presentation experience, and 73.3% had never delivered oral presentations. These numbers reveal a gap between research engagement and translation into scholarly outputs, potentially reflecting limited institutional support for dissemination activities such as publication, seminars, and conferences.

Observing students’ research activities provides further insight into this gap. Most students participated in foundational research tasks such as literature review (62.4%) and proposal writing (62.4%), with fewer involved in downstream analytical activities, including data entry (43.6%) and statistical analyses (26.5%). Although literature review and proposal writing are essential components of the research process, the markedly lower involvement in data analysis and manuscript preparation suggests that students have limited progression through the full research cycle, potentially due to inadequate mentorship and constrained institutional support.

Furthermore, the predominance of cross-sectional studies (42.2%) and case reports (35.5%) reflects a trend toward methodologically simple designs. While it may be appropriate for beginners, these study types offer limited publication potential and hinder the development of advanced research skills. Assar et al. (15) reported similar patterns (38.6% cross-sectional, 23.9% case reports) across six Arab countries. Rjoub et al. (24) found that Palestinian medical students perceived their curricular research education as inadequate (mean adequacy score: 50.3%), particularly in methodology, data analysis, and academic writing. Students with prior research experience showed significantly higher confidence, and a positive correlation was found between perceived adequacy of research training and confidence, suggesting that improved curricula could directly enhance research competencies (24).

Research methodology training appeared relevant to students’ engagement in research projects, with students who had taken these courses showing participation rates of 51.9% versus 24.3% among those who had not completed such courses.

In multivariable logistic regression, academic years, university affiliations, knowledge scores, and total attitude scores were independent predictors of research participation, whereas research methodology coursework and other variables were no longer significant after adjustment. This suggests the apparent effect of research courses may be mediated by students’ knowledge and attitudes rather than the course completion alone, implying that the quality and practical depth of such training may matter more than its mere completion. Notably, participation increased progressively across academic years, with sixth-year students being 22 times more likely to have participated in research projects than second-year students, possibly reflecting cumulative exposure to clinical research environments and growing recognition of the importance of research for career advancement. These findings emphasize the need for early curricular interventions to foster research engagement, rather than relying on passive accumulation of exposure.

Overall, nearly half of the students (49.8%) reported a high burden of research barriers. The most reported barriers were lack of funding (64%), insufficient time due to academic workload (62.1%), universities prioritizing education over research (60.5%), and limited statistical knowledge (60.5%). Notably, the barrier burden differed by academic year, with fourth-year students experiencing significantly fewer obstacles than third- and sixth-year students, likely reflecting differences in academic workload. Fourth-year students may achieve more balance between research and academic demands, whereas earlier years may be limited by inexperience and later years by increased clinical and academic commitments. These barriers closely mirror those reported across the Arab region. In Egypt, Orebi et al. (12) identified funding limitations, time constraints, and insufficient research training in research methods as the most prevalent barriers. Among six Arab countries, Assar et al. (15) reported that lack of laboratory access (68.1%) and prioritization of education over research (66.8%) were the principal obstacles.

Research limitations and obstacles are common in LMICs (8, 25), and are further compounded in Palestine by chronic political instability, severe movement restrictions, frequent academic disruptions, limited access to clinical research sites, constrained inter-institutional collaboration, and restricted participation in regional conferences and training programs that further hinder research engagement. A recent multi-institutional study among 709 Palestinian medical students found that only 37% had received any training in evidence-based medicine, with critical appraisal identified as the weakest skill domain and understanding of advanced methodological concepts remaining limited (26). Furthermore, 80.8% lacked access to paid academic databases, nearly half had rare or absent academic mentorship, and only 29.9% reported availability of research funding for student projects (26). These structural deficits corroborate the barrier profile identified in our study, where lack of funding, insufficient mentorship, and limited access to research resources were among the most commonly endorsed obstacles.

### 4.1 Recommendations

Our findings point to several actionable steps. First, research education should be integrated longitudinally within the medical curriculum, progressing from basic concepts in early years to advanced skills in later years. Second, Institutions should enhance research capacity by providing practical workshops in person and online on research methods, academic writing, and biostatistics (27). Third, dedicated student research funding is crucial. Small grants supporting materials, data collection, or publication fees can remove critical financial barriers. Fourth, strengthening mentorship, including remote supervision, and encouraging early research involvement and peer collaboration can further increase research participation and productivity (27). Finally, institutions should implement ongoing monitoring and evaluation through feedback surveys and tracking of research output to assess the effectiveness of research training and guide future improvements (24).

### 4.2 Strengths and limitations

This study’s main strength lies in its multi-institutional design spanning five major West Bank universities, providing institutional diversity and enabling identification of cross-university patterns in research engagement. The relatively large sample (*N* = 625) and the use of a previously validated instrument enhance measurement quality. However, some limitations warrant mentioning. Convenience sampling, while necessary given ongoing conflict and institutional disruptions, may introduce selection bias. Students who are more digitally active or already interested in research were more likely to participate in the online survey, potentially inflating positive attitudes, overestimating knowledge and underestimating the true burden of barriers. The absence of a calculable response rate due to the open-access recruitment method further limits our ability to quantify this potential selection bias. The cross-sectional design prevents causal inference. Moreover, self-reported data introduces potential recall bias for participation numbers and publications. Finally, barrier assessment reflected perceptions rather than measured objective constraints.

## 5 Conclusion

Palestinian medical students recognize the value of research, but they face substantial knowledge gaps and systemic barriers that limit their engagement and scholarly dissemination. While around 46% participated in research projects, publication rates remained low, with three-quarters never publishing any work. The barriers they face, including funding shortages, time constraints, inadequate infrastructure, skills deficits, and mentorship gaps, reflect challenges common across LMICs that might also be intensified by Palestine’s unique political context. The path forward requires curriculum integration of research education, structured mentorship, protected research time, institutional support and funding, and cultural change positioning research as an integral component of clinical excellence. While challenges are substantial, Palestinian medical students’ positive stance toward research provides grounds for optimism. Palestine’s healthcare future depends on training not just clinically competent physicians but a research-literate workforce capable of addressing local and regional health challenges and generating scientific evidence.

## Data Availability

The raw data supporting the conclusions of this article will be made available by the authors, without undue reservation.
