# Supplementary material for: Undergraduate medical research in the West Bank, Palestine: a cross-sectional analysis of student knowledge, attitudes, barriers, and engagement across five universities
Source: Front Med (Lausanne). 2026 May 1;13:1803680. doi: 10.3389/fmed.2026.1803680 (PMC13175854; doi:10.3389/fmed.2026.1803680)
Supplement: Supplementary file 1 [file Supplementary_File_1.DOCX]

**Table 1. Number and Percentages of Participants Reporting Each Research Barrier.**

| **Barrier** | **N (%)** |
| --- | --- |
| 1-Lack of suitable research space | 344 (55) |
| 2-Dissatisfaction with the encouragement of researchers to do research | 299 (47.8) |
| 3-Poor attention is given to researchers and creative faculty | 314 (50.2) |
| 4-Priority on education over research in the university | 378 (60.5) |
| 5-Lack of time to do research because of educational tasks | 388 (62.1) |
| 6-Poor collaboration between departments and research centers | 317 (50.7) |
| 7-Personal economic & financial problems | 288 (46.1) |
| 8-Insufficient research skills | 347 (55.5) |
| 9-Lack of familiarity with research studies | 350 (56) |
| 10-Lack of familiarity with statistical analysis | 378 (60.5) |
| 11-Lack of skills for writing papers | 327 (52.3) |
| 12-Lack of skills for submitting articles | 342 (54.7) |
| 13-Lack of familiarity with research proposal writing | 319 (51) |
| 14-Lack of good research ideas | 280 (44.8) |
| 15-Lack of ability to publish an article after the completion of the project | 311 (49.8) |
| 16-Boring and difficult to research because of the lack of skill | 277 (44.3) |
| 17-Lack of research needs and priorities in the university health system | 289 (46.2) |
| 18-Lack of coordination of research priorities with research ideas | 283 (45.3) |
| 19-Inappropriate or insufficient consultation before drafting research proposals | 281 (45) |
| 20-Inappropriate or insufficient guidance for writing | 316 (50.6) |
| 21-Lack of research applications in personal life and professional jobs | 293 (46.9) |
| 22-Lack of interest in research | 224 (35.8) |
| 23-Lack of significant income to do research | 292 (46.7) |
| 24-Lack of confidence in the potential for completing research | 288 (46.1) |
| 25-Prefer to use the free time to do other tasks | 348 (55.7) |
| 26-Fear of making mistakes in research and being blamed by others | 293 (46.9) |
| 27-Lack of professor input with students | 328 (52.5) |
| 28-Lack of timely funding of research & lack of funds | 400 (64) |
| 29-Lack of appropriate databases | 307 (49.1) |
| 30-Lack of access to laboratory equipment for performing a research project | 329 (52.6) |
| 31-Lack of access to studies across the country | 331 (53) |
| 32-lack of cooperation between research centers | 331 (53) |

N (%) represent participants who agreed with each barrier statement

**Table 2. Comparison of Knowledge Score, Attitude Subscales Scores, and Barrier Burden Score Based on Gender and Parental Educational Levels.**

| **Score** | **Gender** | | **P-value** | **Father’s Educational Status** | | **P-value** | **Mother’s Educational Status** | | **P-value** |
| --- | --- | --- | --- | --- | --- | --- | --- | --- | --- |
|  | **Male** | **Female** |  | **Tertiary level** | **Non-Tertiary level** |  | **Tertiary level** | **Non-Tertiary level** |  |
| **Knowledge Score** | 4 [2 – 6] | 4 [2 – 6] | 0.532 | 4 [2 – 6] | 3 [1.75 – 5] | **0.034*** | 3 [2 – 6] | 4 [2 – 6] | 0.909 |
|  | *308.74* | *317.71* |  | *323.53* | *290.95* |  | *313.57* | *311.83* |  |
| **Research Usefulness** | 25 [21 – 26] | 25 [22 – 27] | **0.044*** | 25 [22 – 26] | 25 [22 – 26] | 0.412 | 25 [22 – 26] | 25 [22 – 26] | 0.804 |
|  | *299.34* | *328.09* |  | *317.05* | *304.53* |  | *314.24* | *310.46* |  |
| **Anxiety Score** | 15 [13 – 18] | 15 [12 – 18] | 0.201 | 15 [12 – 18] | 15 [12 – 18] | 0.287 | 15 [12 – 18] | 15 [12 – 18] | 0.576 |
|  | *321.76* | *303.32* |  | *318.30* | *301.90* |  | *315.81* | *307.23* |  |
| **Positive Attitude** | 18 [14 – 20] | 19 [15 – 21] | **0.040*** | 18 [14 – 21] | 17 [14 – 20.25] | 0.242 | 18 [14 -21] | 18 [15 – 20] | 0.721 |
|  | *299.04* | *328.42* |  | *318.77* | *300.91* |  | *311.22* | *316.64* |  |
| **Research Relevance** | 8 [8 – 10] | 9 [8 – 10] | 0.103 | 8 [8 – 10] | 9 [8 – 10] | 0.982 | 8 [8 – 10] | 9 [7 – 10] | 0.955 |
|  | *301.97* | *325.18* |  | *312.89* | *313.24* |  | *312.72* | *313.57* |  |
| **Difficulty Score** | 6 [5 – 7] | 6 [5 – 7] | 0.863 | 6 [5 – 7] | 6 [5 – 7] | 0.990 | 6 [5 – 7] | 6 [5 – 7] | 0.662 |
|  | *314.17* | *311.71* |  | *312.94* | *313.13* |  | *315.17* | *308.56* |  |
| **Barriers Burden score** | 76 [65 – 85] | 75 [64 – 85] | 0.558 | 76 [65 – 86] | 73 [64 – 84] | 0.065 | 75 [64 – 86] | 76 [65 – 85] | 0.863 |
|  | *317.02* | *308.56* |  | *322.22* | *293.69* |  | *312.13* | *314.79* |  |

For each score, the first row presents Median [Q1 -Q3] and the second row (*in italics*) presents Mean Rank. P-values derived from Mann-Whitney U tests. **Bold values** indicate statistical significance (*p < 0.05).

**Table 3. Comparison of Knowledge Score, Attitude Subscales Scores, and Barrier Burden Score Based on Research-Related Activities.**

| **Score** | ***Have you studied research methodology at university?*** | | ***P-value*** | ***Have you enrolled in a research workshop or training*** | | ***P-value*** | ***Have you participated in a research project?*** | | ***P-value*** |
| --- | --- | --- | --- | --- | --- | --- | --- | --- | --- |
|  | **Yes** | **No** |  | **Yes** | **No** |  | **Yes** | **No** |  |
| **Knowledge Score** | 4 [2 – 6] | 2.5 [1- 5] | **< 0.001*** | 5 [3 – 6] | 3 [1 – 5] | **< 0.001*** | 4 [3 – 6] | 3 [1 – 5] | **< 0.001*** |
|  | *330.10* | *251.51* |  | *356.49* | *278.83* |  | *356.06* | *276.44* |  |
| **Research Usefulness** | 25 [22 – 26] | 25 [22 – 26] | 0.519 | 25 [23 – 27] | 24 [21 – 26] | **< 0.001*** | 25 [23 – 27] | 25 [21 – 26] | **0.003*** |
|  | *310.57* | *321.72* |  | *347.71* | *285.73* |  | *336.24* | *293.27* |  |
| **Anxiety Score** | 15 [12 – 18] | 15.50 [13 – 17] | 0.843 | 16 [12 – 19] | 15 [12 – 17] | **0.050*** | 16 [12 – 19] | 15 [12 – 17] | **0.044*** |
|  | *312.25* | *315.71* |  | *328.93* | *300.48* |  | *328.75* | *299.63* |  |
| **Positive Attitude** | 18 [14 – 21] | 19 [14 – 21] | 0.342 | 20 [15 – 21] | 17 [14 – 20] | **< 0.001*** | 19 [15 – 21] | 18 [14 – 20] | **0.003*** |
|  | *309.43* | *325.85* |  | *354.57* | *280.34* |  | *336.09* | *293.39* |  |
| **Research Relevance** | 9 [8 – 10] | 8 [7.25 – 10] | 0.324 | 9 [8 – 11] | 8 [7 – 9.25] | **< 0.001*** | 9 [8 – 11] | 8 [7 – 10] | **< 0.001*** |
|  | *316.70* | *299.69* |  | *364.99* | *272.15* |  | *349.87* | *281.69* |  |
| **Difficulty Score** | 6 [5 – 7] | 6 [5 – 7] | 0.517 | 6 [5 – 7] | 6 [4 – 7] | **< 0.001*** | 6 [5 – 7] | 6 [4 – 7] | **0.015*** |
|  | *315.43* | *304.26* |  | *350.51* | *283.53* |  | *331.85* | *297.00* |  |
| **Barriers Burden score** | 76 [65 – 85] | 75 [64 – 86.75] | 0.596 | 75 [65 – 84] | 76 [65 – 86] | 0.184 | 75 [64 – 86] | 76 [65 – 84.25] | 0.468 |
|  | *310.98* | *320.26* |  | *302.18* | *321.50* |  | *307.31* | *317.83* |  |

For each score, the first row presents Median [Q1 -Q3] and the second row (*in italics*) presents Mean Rank. P-values derived from Mann-Whitney U tests. **Bold values** indicate statistical significance (*p < 0.05).
